# Mid-term evaluation of perioperative i.v. corticosteroid treatment efficacy on overall and audiological outcome following CO_2_ laser stapedotomy: a retrospective study of 84 cases

**DOI:** 10.1007/s00405-020-05816-z

**Published:** 2020-01-28

**Authors:** L. Székely, A. Gáborján, K. Dános, T. Szalóki, Z. Fent, L. Tamás, G. Polony

**Affiliations:** Department of Oto-Rhino-Laryngology, Head and Neck Surgery, Semmelweis University, Szigony Str. 36, Budapest, 1083 Hungary

**Keywords:** Stapes surgery, Corticosteroid therapy, Otosclerosis, Stapedotomy, CO_2_ laser, Middle ear, Audiology

## Abstract

**Purpose:**

Our aim was to determine whether perioperatively administered corticosteroid treatment has any beneficial effect on the outcome of stapes surgery, with special regard to the audiological results and early postoperative morbidity.

**Methods:**

84 CO_2_ laser stapedotomies performed in our institute between 2013 and 2018 were included in our investigation. All cases underwent preoperative and mid-term postoperative pure-tone audiometric evaluation. Vestibular complications were also evaluated. The cases were subdivided into two groups, 23 patients received perioperative i.v. methylprednisolone treatment (“S”) while the other 61 patients (“nS”) did not receive any adjuvant pharmacological therapy. The data were analyzed retrospectively using IBM SPSS Statistics.

**Results:**

CO_2_ laser stapedotomy proved to be a successful intervention with a significant improvement in ABG and AC thresholds as well. Long-term BC levels were significantly better compared to preoperative ones in the S group; however, in the nS group, no difference could be shown. Hearing and ABG gain were significantly superior in group S [28.1 dB (SD11.2) vs. 18.1 dB (SD 10.9) and 23.9 dB(SD 9.8) vs. 17.2 dB (SD 9.5), respectively].

**Conclusion:**

No significant inner ear damage was detectable in the results of our CO_2_ laser stapedotomy method; however, the positive effect of corticosteroid treatment could be demonstrated through the postoperative hearing levels. We found no statistical difference in early postoperative morbidity. According to our data, the routine administration of corticosteroids during stapes surgery could be an issue worthy of consideration. The effects of perioperative treatment vs that on the first day after surgery, and topical vs. systemic treatment could be the subject of further investigation in a prospective manner.

## Introduction

Since the description of the surgical treatment of stapes footplate fixation by Shea in 1956 [1], the technique underwent numerous minor and major changes. Nowadays, stapes surgery is considered to be a relatively safe and efficient surgical procedure. Stapedotomy definitely led to better audiological results and fewer complications [2] compared to the original method, stapedectomy. Further improvements could be observed with the introduction of laser-stapedotomy [3, 4]; however, not enough data are available to draw a distinction between the outcomes of the different laser techniques. According to available data, CO_2_ and KTP laser are superior compared to microdrill and other laser techniques [5]. Each of the above-mentioned developments in the surgical technique resulted from the urge to decrease the risk of causing inner ear damage, being the cause of SNHL and vestibular dysfunction, the major complications after stapes surgery. Usually, these complications result from the mechanical or thermal trauma to the ossicular chain, especially the stapes footplate, and to the vestibulum of the middle ear. The effectiveness of corticosteroids in acute inner ear damage has already been shown, but considering the numerous possible side effects, the administration of these drugs requires individual evaluation based on solid evidence. Our hypothesis was that perioperatively administered corticosteroid treatment could have a protective effect against intraoperative inner ear damage, thus audiological outcome could be superior, and the number of complications could be lower compared to the control setting. The aim of this study was to evaluate the possible beneficial effect of perioperative corticosteroid treatment on the outcome of stapes surgery, with special regard to the audiological results and early postoperative morbidity.

## Materials and methods

### Patients

A retrospective study of 84 cases of CO_2_ laser stapedotomy was performed. Between 2013 and 2018, 101 primary CO_2_ laser-assisted stapedotomies were carried out at the Department of Oto-Rhino-Laryngology, Head and Neck Surgery Semmelweis University, Budapest. All cases included were primary middle ear surgeries on the affected side. 17 patients were excluded due to the postoperative follow-up data being incomplete. The presumed preoperative clinical diagnosis of stapes fixation was made by PTA testing, tympanometry, stapedius reflex, and physical examination. 29 patients were male and 55 were female out of the 84 cases, with an average age of 46.52 (SD 12.09) years.

### Surgical technique

In all the above cases, the same standardized surgical method was applied and was performed by the same senior surgeon. After an endural approach, the posterior TM flap was lifted, and the stapes fixation was verified by the palpation of the ossicular chain. After the sharp dissection of the ISJ, the posterior crus of the stapes was vaporized with CO_2_ laser single shot technique (OPAL L30; TrueScan scanner; UniMax 2000 micromanipulator; Lasram, Budapest, Hungary), and the superstructure was removed in one piece by downfracturing the anterior crus. Stapedotomy was performed exclusively with CO_2_ laser vaporization (21 W, 0.1 s). The fenestra diameter was 0.6 mm or 0.8 mm according to the prosthesis diameter. The prosthesis loop was manually placed or crimped on the long process of the incus in 83 cases; in one case, malleovestibulopexy was performed. The prosthesis loop was bridged with a small connective tissue to prevent slipping and necrosis of the long process. The TM flap was restored, meatal incisions were overlaid with sterile silicone sheets, and the ear canal was stuffed with ofloxacin-impregnated absorbable hemostatic gelatin sponge (Spongostan) pieces. The incision was closed with sutures.

The applied prosthesis length was 4.5 mm in 95.2% of the cases, with a diameter of 0.4 mm in 15.5% and 0.6 mm in 84.5%. The majority (85.4%) of the applied pistons were Schuknecht fluoroplastic, stainless, slim shaft wire pistons, diameter 0.6 mm (Gyrus Acmi, USA), Titanium Soft Clip Stapes Pistons, diameter 0.6 mm (Kurz, Germany) were used in 6.1%, and Richards Platinum Fluoroplastic Pistons, diameter 0.4 mm (Olympus, Japan, (formerly Gyrus)) were applied in 8.5%. The type of prosthesis was selected according to the availability.

## Methods

The cases were subdivided into two groups: in the first group (“S”, *n* = 23), patients received perioperative i.v. methylprednisolone, while patients in the second group (“nS”, *n* = 61) did not receive any steroids perioperatively. The patients in the steroid group were selected on an ad hoc basis. Female-to-male ratio was 14/9 and 41/20, the average age at the time of surgery was 46.9 (SD 10.5) vs 46.4 (SD 12.7) years, respectively.

In the steroid group, patients received 500 mg methylprednisolone i.v. during the first 2 h after the beginning of the surgery. The audiological outcome was analyzed based on preoperative and mid-term postoperative pure-tone audiometric (PTA) evaluation, in 70 cases, pure-tone bone conduction was also registered on the first postoperative day to rule out any inner ear damage. The average postoperative follow-up time was 175 days (SD 216.8; range 29–1104), which could be considered as a reliable indicator of the long-term results [6]. The AC levels were measured at 0.125; 0.25; 0.5; 1; 2; 4; 8 kHz, and the BC were registered at 0.25; 0.5; 1; 2;4 kHz. Evaluation was done according to the Committee on Hearing and Equilibrium guidelines of the American Academy of Otolaryngology Head and Neck Surgery (AAO-HNS) [7]. The thresholds were reported, and the extended pure tone average was calculated from 0.5; 1; 2; 3 kHz frequencies. The 3 kHz values were considered as the mean of 2 and 4 kHz [8]. The ABG was calculated from same-time measurements. Vestibular complications and the number of days spent in the hospital were also noted. The duration of vestibular symptoms (nausea, vomiting, nystagmus) was reported in hours. These data were retrospectively analyzed statistically (IBM SPSS v. 25, Armonk, NY, USA) to define the results of stapedotomies and the effect of corticosteroid treatment. Independent Samples *T* test was used for analyzing group differences, while intervention success was examined with Paired Samples *t* test. The statistical significance level was *p* > 0.05.

## Results

The group characteristics are presented in Table 1. The age and sex distributions were similar across the two groups. According to the preoperative evaluation, the average hearing thresholds were, AC-PTA 53.9 dB (SD 14.2; range 20.6–95 dB) BC-PTA 24.8 dB (SD 11; range 5.6–56.9 dB), ABG-PTA 29.05 dB (SD 8.21; range 12.5–48.1 dB). 88% of the cases had a 20 dB or higher ABG, and a functional hearing (AC ≤ 30 dB) could only be detected in two cases. No significant difference could be revealed regarding the preoperative hearing thresholds and follow-up lengths between the groups.

**Table 1:** Group characteristics of 84 stapes surgery cases

		Groups		Significance	
		S (*n* = 23)	nS (*n* = 61)		
preop. BC-PTA (dB, mean ± SD)		26.19 ± 12.8	24.30 ± 10.43	*p* = 0.491	ns
preop. ABG-PTA (dB, mean ± SD)		31.82 ± 8.35	28.0 ± 7.98	*p* = 0.057	ns
preop. AC-PTA (dB, mean ± SD)		58.14 ± 17.05	52.30 ± 12.77	*p* = 0.093	ns
age (years, mean ± SD)		46.91 ± 10.51	46.38 ± 12.70	*p* = 0.857	ns
follow up time (days, mean ± SD)		159.08 ± 203.43	218.13 ± 248.62	*p* = 0.268	ns
gender (female/male ratio)		1.556	2.050	*p* = 0.586	ns
Piston used	Kurz Soft Clip	4%	7%		
	Richards	22%	7%		
	Schucknecht Wire	74%	87%		

**Fig. 1:**
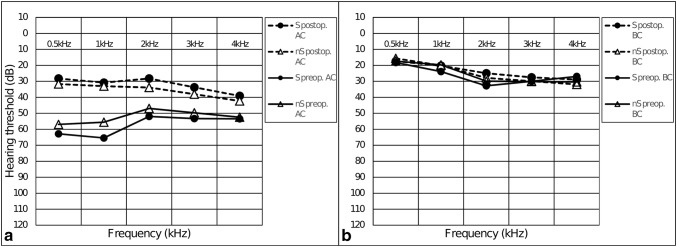
**a** Pre- and postoperative pure tone audiometry air conduction hearing thresholds. Significant improvement after surgery in both groups. **b** Pre- and postoperative pure tone audiometry bone conduction hearing thresholds. No significant sensorineural hearing loss could be detected

**Fig. 2:**
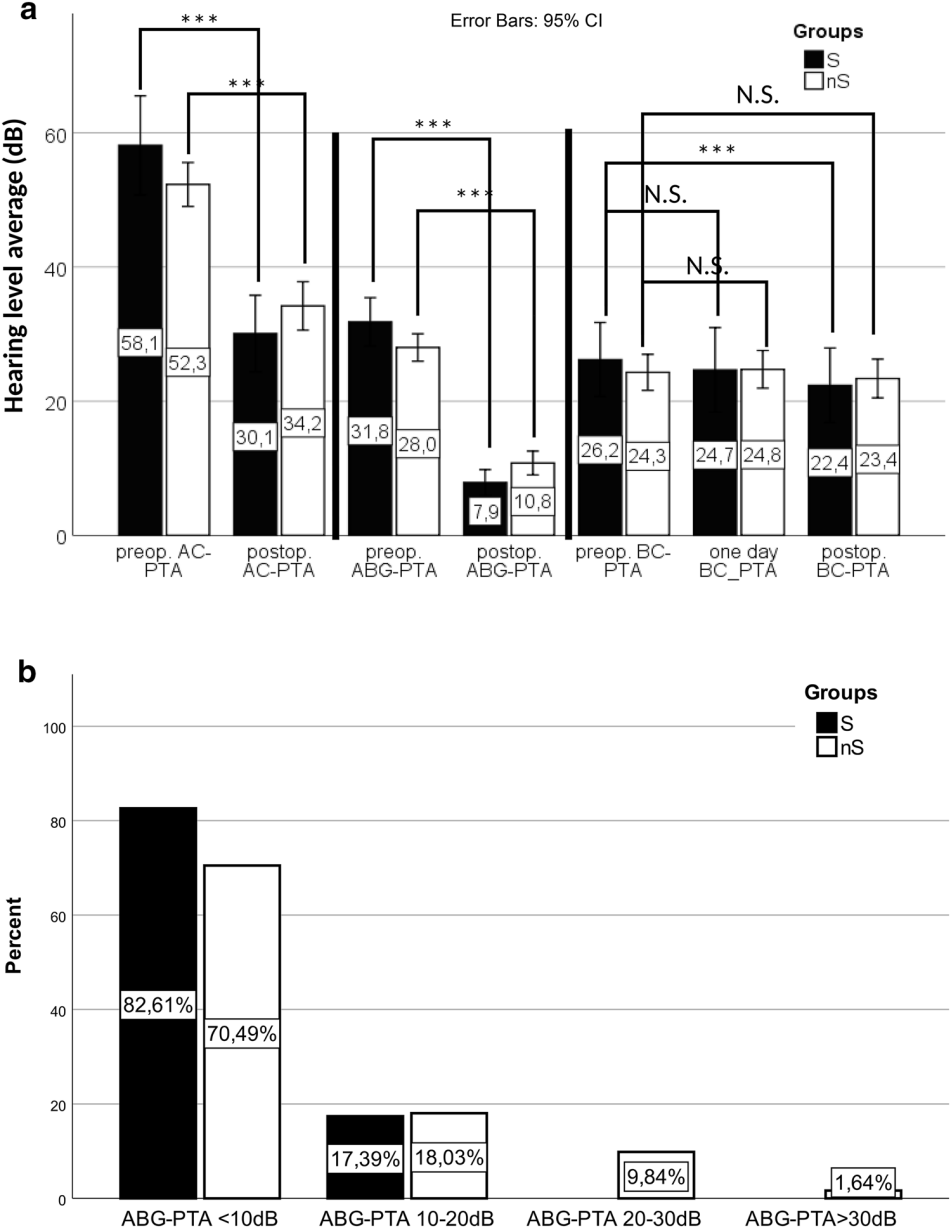
Extended pure tone average measurements. **a** Evolution of AC, ABG, and BC levels after surgery. **b** Distribution of postoperative ABG-PTAs of each group in 10 dB bins

Average post-surgery AC-PTA was 33.07 dB (SD 13.9). AC-PTA significantly improved after the surgical intervention in both, S group: 58.14 dB (SD 17.0) vs. 30.08 dB (SD 13.2) (*p* < 0.001); and nS group: 52.31 (SD 12.8) vs. 34.20 dB (SD 14.1) (*p* < 0.001) (Figs.1a, 2a). The minimum of 30 dB AC-PTA was achieved in 52.4% of the cases (S group 52%; nS group 52.5%). After the surgery, the mean ABG-PTA was 10.0 dB (SD 6.5). The ABG closure ≤ 10 dB was gained in 82.6% and 70.5%, ABG closure between 10 and 20 dB prevailed 17.4% and 18.0% in S and nS groups, respectively, 11.5% of the nS group had ABG-PTA > 20 dB (Fig. 2b). The postoperative ABG-PTA differed significantly from the preoperative ABG-PTA in both S (*p* < 0.001) and nS (*p* < 0.001) groups with a mean improvement of 23.9 dB and 17.2 dB, respectively (Fig. 2a).

BC-PTA on the first day after surgery did not differ from the preoperative thresholds in S group (*p* = 0.300), but the long-term BC-PTA level showed significant improvement [26.19 dB (SD 12.8) vs 22.37 dB (SD 12.8); *p* = 0.001] (Fig. 2a). In the nS group, no statistically significant difference could be seen comparing the preoperative and the first day after surgery (*p* = 0.208) or longer term (*p* = 0.223) postoperative BC-PTA levels (Fig. 2a).

**Fig. 3:**
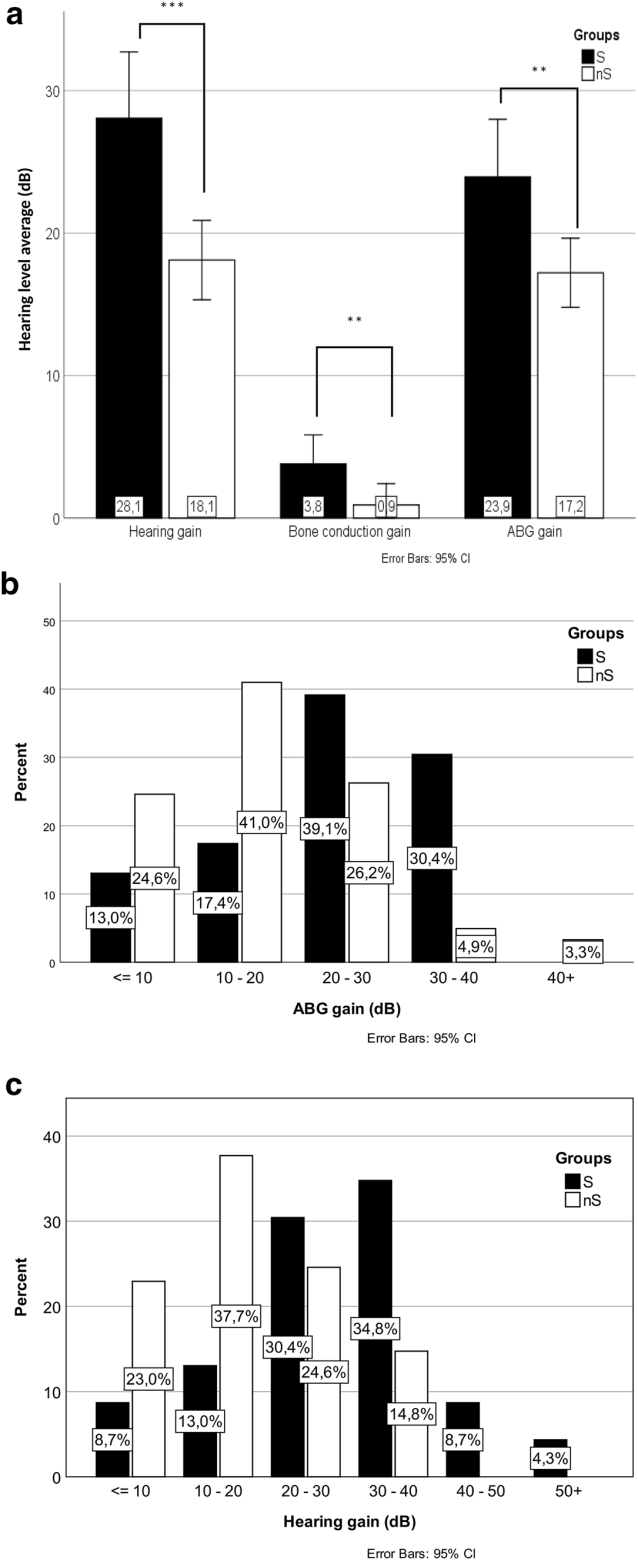
Postoperative audiological improvement is shown across groups (**a**), concerning hearing gain, ABG gain and bone conduction gain (**b**). Distribution of ABG improvement (**c**) and AC improvement in 10 dB bins.

**Fig. 4:**
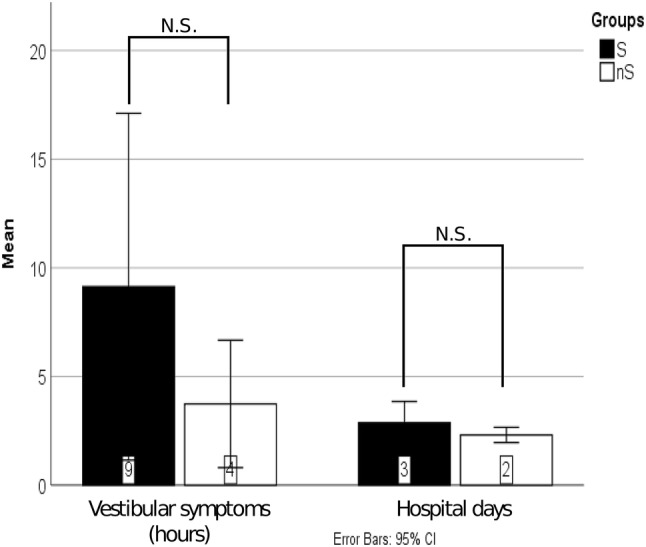
Postoperative morbidity: average time of vestibular dysfunction, and length of hospitalization

Hearing gain (defined as a difference between preoperative and postoperative AC-PTA) was significantly different in the two groups (*p* < 0.001) with a mean improvement of 28.1 dB (SD 11.2) and 18.1 dB (SD 10.9), while the difference in ABG gain across groups of S and nS was also significant (*p* = 0.005) 23.9 dB (SD 9.8) vs. 17.2 dB (SD 9.5), respectively (Fig. 3a). In the S group, 69.6% had an ABG gain > 20 dB, compared to the nS group, where it was 34.4% (Fig. 3c). The percentage of hearing gain > 20 dB was 82.6% S vs 39.3% nS group (Fig. 3b). Mean BC gain was significantly higher (*p* = 0.038) in the S group 3.81 dB (SD 4.8) compared to the nS group 0.92 dB (SD 5.8) (Fig. 3a). No significant difference was discovered concerning vestibular complications and hospital days between the two groups (*p* = 0.215; *p* = 0.182) (Fig. 4).

## Discussion

### Primary findings

CO_2_ laser stapedotomy is an efficient treatment option for stapes fixation, according to AAO-HNS guidelines (surgical success is defined by postoperative ABG of 10 dB or less [7]) high success rates were seen in both series. The primary presumption of this result was that corticosteroids could have a positive impact on sensorineural hearing thresholds through inner ear (cochlea) protective mechanisms [9, 10]. Although no significant inner ear damage was detected in the results of our stapedotomy series, the S group had significantly better results compared to the nS group, considering hearing gain, ABG gain, and bone conduction gain. Moreover, in the S group, postoperative BC-PTA levels showed significant improvement, while there was no such difference in the nS group. Looking at the BC-gain and ABG-gain data, it is apparent that the main reason behind this finding is the higher ABG improvement of the S group. Compared to the 20 dB average AC improvement in stapes surgery, both differences of 2.9 dB (14.5%) in BC gain and 6.7 dB (33.5%) in ABG-gain across the groups must be considered as clinically relevant. According to our data, perioperative corticosteroid has a positive effect on audiological outcomes, not only affecting the sensorineural thresholds, but mainly through the contribution to a better ABG closure. No effect on postoperative complications could be discovered.

## Limitations

The less-than-1-year average postoperative follow-up could be a limitation by excluding the impact of long-term complications such as incus necrosis or prosthesis dislocation. However, no effect of corticosteroid treatment is anticipated concerning these conditions, therefore this circumstance has no distortional influence. Although the group characteristics were statistically not different, the patients were selected on an ad hoc basis, which could lead to potential selection bias. The data represent the results of a single institution and a single surgeon. Evaluation is based exclusively on subjective hearing testing; however, pure-tone audiometry is still considered as the gold standard for monitoring hearing outcomes after middle ear surgery.

Surgeries were performed with three different types of pistons. We suspect no distortions concerning the usage of Soft Clip pistons, since it was used in the same proportion and in minor amount of the cases (1 in S, 4 in nS).

In both groups, the majority of the surgeries were performed with Richards and Schuknect type pistons. Both pistons are manufactured by the same company (formerly Gyrus ACMI acquired by Olympus) and have identical fluoroplastic shafts. Richards is a second-generation piston, differences between the two designs are in the material and shape of the loop (platinum vs stainless steel, flat vs round cross-section, respectively). No direct comparison could be found in the literature, but it has been shown that Schunknect type piston is not inferior to other second generation pistons and has comparable results [11].

Concerning the diameter of pistons, it looks like there is no significant difference in audiological results between 0.6 mm and 0.4 mm; however, it still could be a subject of discussion [12].

### Interpretation of the other results

To date, there is limited knowledge about the efficacy of corticosteroid treatment on laser stapedotomy outcome. We found a positive effect of perioperative corticosteroid therapy on stapes surgery hearing results. While this is in contrast with the findings of other clinical investigations [13, 14] the beneficial inner ear effect of corticosteroids in ASNHL has been proven already [15], furthermore, multiple non-clinical studies have shown a protective effect in case of cochlear trauma and otologic procedures [9, 10, 16]. Methylprednisolone has a protective inner ear effect by reducing vestibular trauma and electrode impedances after cochlear implantation [17]. According to our data and the results of the above authors, perioperative CS administration has a cochlea-protective effect during laser stapedotomy. Interestingly enough, the cochlear effect played a minor role in the better hearing gain of the S group, whereas better ABG closure proved to be the major reason behind it. While no such observation exists in the literature, looking at the various physiological effects of methylprednisolone, there are several explanations for this phenomenon. Postoperative ABG represents the function of the middle ear and the implanted piston. According to our experience and that of other authors, in revision stapes surgery [18], movement of the piston is frequently compromised by narrowing of the stapedotomy hole, or by reactive soft tissue formation (periprosthetic fibrosis, granulation tissue, adhesions) in the cavum tympani. The development of intratympanic scar tissue could also lead to impaired Eustachian tube function and middle ear ventilation, compromising middle ear performance. Experimentally, methylprednisolone has a dose-dependent inhibition effect on granulation tissue growth [19], and corticosteroids have a positive effect on adhesive inflammations by decreasing the amount of vascular hyperplasia and fibrosis [20]. Corticostreoids are used on different surgical fields to control postoperative scar tissue formation. Triamcinolone decreases postoperative fibrin formation after cataract surgery [21]. Methylprednisolone prevents epidural fibrosis [22] and cauda equina adhesions [23] after neurosurgical procedures, while also having a positive impact on postoperative pericardial adhesion formation [24]. Summarizing these observations, it is possible that the positive effect of periopertive high dose i.v. methylprednisolone on ABG closure is caused by the inhibition of uncontrolled periprosthetic scar tissue formation. Further investigation is needed for the explanation of these findings, including the evaluation of the efficacy of intraoperative intratympanic corticosteroid treatment. The side effects of systemic corticosteroid treatment could be a limitation of its routine administration in stapes surgery. These adverse effects include elevated blood sugar levels, elevated blood pressure, psychological disturbances, weight gain, and gastrointestinal problems, which usually resolve following the end of therapy. In our practice, perioperative treatment consisted of only one dose of i.v. methylprednisolone, therefore only acute effects were anticipated. During the average 2-day-long hospitalization, there were no serious problems reported concerning blood sugar and blood pressure values, nor other corticosteroid-associated adverse reactions. The use of topical corticosteroids would further decrease the chance of undesirable systemic effects, while higher intracochlear and intratympanic concentrations could be achieved. According to these data, the administration of methylprednisolone in stapes surgery could be considered a relatively safe procedure for maximizing audiological results. Still, more investigation is needed to clarify the effects of routine administration. Short-term postoperative BC measurement is a reliable method for identifying surgery-related acute inner ear damage. BC testing is possible from the first day following the surgery, as meatal obstruction (packaging, inflammation, blood clot, etc.) does not influence results. In case of no prophylactic corticosteroid treatment, the use of short-term BC levels are becoming more important to identify and indicate treatment in time. However, these measurements take place on the first day following the surgery, thus, by using this method, the time elapsing between the moment of the trauma and the beginning treatment could increase by 24 h or more. The efficacy of perioperative treatment vs that on the first day after the surgery, and the effectiveness of topical vs. systemic treatment should be subject to further investigation.

## Abbreviations

TM: Tympanomeatal
ISJ: Incudostapedial joint
AC: Air conduction
BC: Bone conduction
ABG: Air–bone gap
PTA: (Extended) pure tone average
S: First group: patients received corticosteroid perioperatively
nS: Second group: patients did not received any corticosteroid perioperatively
